# Surgical Resection and Abdominal Wall Reconstruction of a Rapidly Progressive Desmoid Tumor: A Case Report

**DOI:** 10.7759/cureus.85911

**Published:** 2025-06-13

**Authors:** Miguel Jiménez-Yarza, Manuel Landeros-Muñoz, Angela L Juárez-Villarreal, Martín S Rodríguez-Garza, José D Ortiz-Cisneros

**Affiliations:** 1 General Surgery, Institute for Social Security and Services for State Workers (ISSSTE), Monterrey, MEX; 2 Surgical Oncology, Institute for Social Security and Services for State Workers (ISSSTE), Monterrey, MEX

**Keywords:** abdominal wall, case report, desmoid tumor, mesh repair, reconstruction

## Abstract

Desmoid tumors (DTs) are rare, locally invasive soft tissue neoplasms characterized by fibroblastic proliferation and a lack of metastatic potential. Despite their histologically benign nature, their infiltrative behavior and unpredictable progression present significant therapeutic challenges, particularly when located in the abdominal wall, where resection and reconstruction may be necessary. We present the case of a 37-year-old woman with a rapidly enlarging, painful mass in the right rectus abdominis muscle. Imaging and biopsy confirmed desmoid-type fibromatosis. After a period of surveillance, the tumor doubled in size over six months, necessitating surgical excision. A full-thickness resection resulted in a 15×21 cm abdominal wall defect, which was reconstructed using Sepramesh mesh anchored to both peritoneum and fascia, followed by layered closure. Histopathology revealed a spindle-cell neoplasm infiltrating skeletal muscle with negative surgical margins. At eight months postoperatively, the patient remained asymptomatic and free of recurrence. This case underscores the importance of individualized treatment strategies in desmoid tumors, particularly in the abdominal wall, where reconstruction plays a critical role. Mesh-reinforced repair appears to be a safe and effective option, as supported by recent literature. Surgical resection with negative margins and appropriate reconstruction can achieve durable outcomes, emphasizing the need for long-term surveillance.

## Introduction

Desmoid tumors (DTs), also known as aggressive fibromatosis or desmoid-type fibromatosis, are rare, locally invasive neoplasms arising from musculoaponeurotic structures, characterized by monoclonal fibroblastic proliferation and a tendency for infiltrative growth without metastatic potential. They account for approximately 0.03% of all neoplasms and less than 3% of soft tissue tumors, with an estimated annual incidence ranging from two to six cases per million people worldwide [1-3].

DTs typically present between the ages of 30 and 40 years, with a clear female predominance, particularly among women of childbearing age. Hormonal influences, notably estrogen, are thought to contribute to this sex disparity, as evidenced by increased incidence during pregnancy and responsiveness to antiestrogen therapy such as tamoxifen [2,4,5].

Clinically, desmoid tumors exhibit a highly variable and often unpredictable course. Many are discovered incidentally through imaging, while others present with symptoms secondary to local mass effect, including pain, deformity, or functional impairment due to compression or invasion of adjacent organs and structures. Intra-abdominal DTs, frequently associated with familial adenomatous polyposis (FAP) or Gardner syndrome, can lead to serious complications such as bowel obstruction or ischemia. Approximately 25% of sporadic DTs are associated with prior surgical trauma, and 85-90% harbor somatic mutations in the CTNNB1 gene, encoding β-catenin [2,3,6].

Desmoid tumors are broadly categorized into abdominal and extra-abdominal types. Abdominal DTs include superficial (abdominal wall) and intra-abdominal forms, the latter often related to FAP and typically involving the mesentery or retroperitoneum. In contrast, sporadic DTs are more commonly extra-abdominal and present as solitary lesions [1,7].

Imaging plays a pivotal role in diagnosis, staging, and monitoring. Magnetic resonance imaging (MRI) is the modality of choice due to its superior soft tissue resolution and ability to delineate tumor margins and involvement of adjacent structures. Computed tomography (CT) and ultrasonography are also utilized, particularly for anatomical assessment, surgical planning, and evaluation of complications [1-3].

Historically, surgical resection was the cornerstone of treatment; however, due to high recurrence rates and considerable morbidity, current guidelines favor an initial strategy of active surveillance, especially for asymptomatic or stable lesions. This approach is supported by evidence showing spontaneous regression in up to 20% of cases, while 50% remain stable and only a minority exhibit rapid progression. Management should prioritize pain control and preservation of quality of life, avoiding overtreatment when possible [8,9]. This case highlights the rare scenario of a rapidly enlarging abdominal wall DT requiring extensive resection and complex reconstruction. It offers practical insights into decision-making when balancing oncologic control with abdominal wall integrity and cosmetic outcome.

## Case presentation

**Figure 1:**
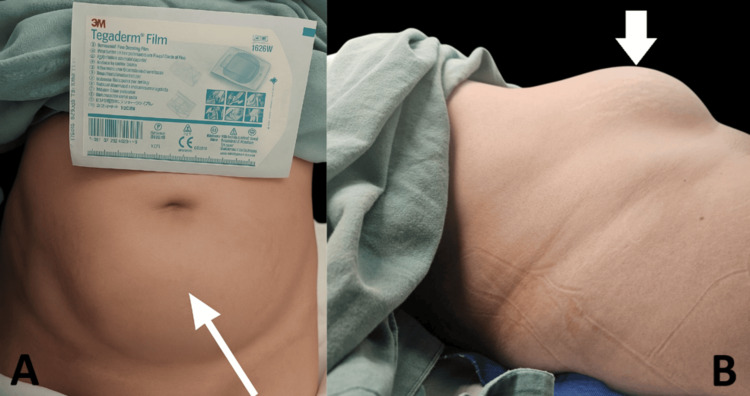
Clinical image of the patient preoperatively (A) Frontal view showing a prominent, well-circumscribed lower abdominal mass (white arrow) causing outward bulging of the abdominal wall. A Tegaderm™ Film dressing is placed for scale; (B) Lateral view of the same mass, highlighting its projection and subcutaneous extension (white arrow). The arrow indicates the area of maximum distension consistent with the tumor.

**Figure 2:**
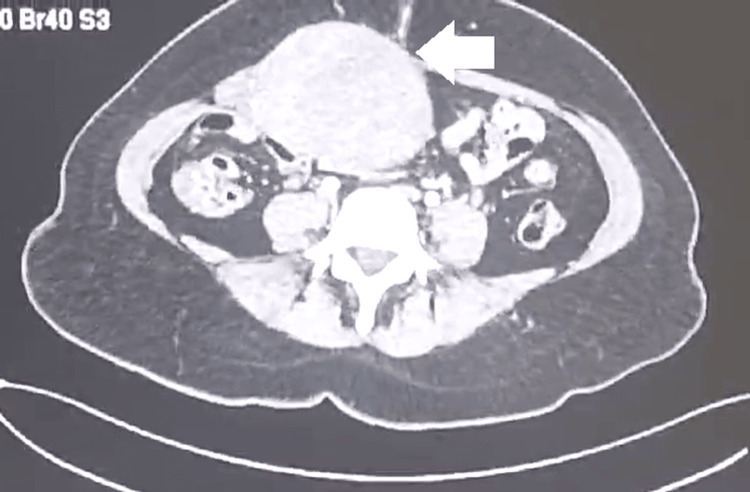
Axial CT image during the arterial phase The scan reveals a well-defined soft tissue mass located in the anterior abdominal wall. The white arrow indicates the central portion of the lesion, showing heterogeneous density and displacement of adjacent structures.

**Figure 3:**
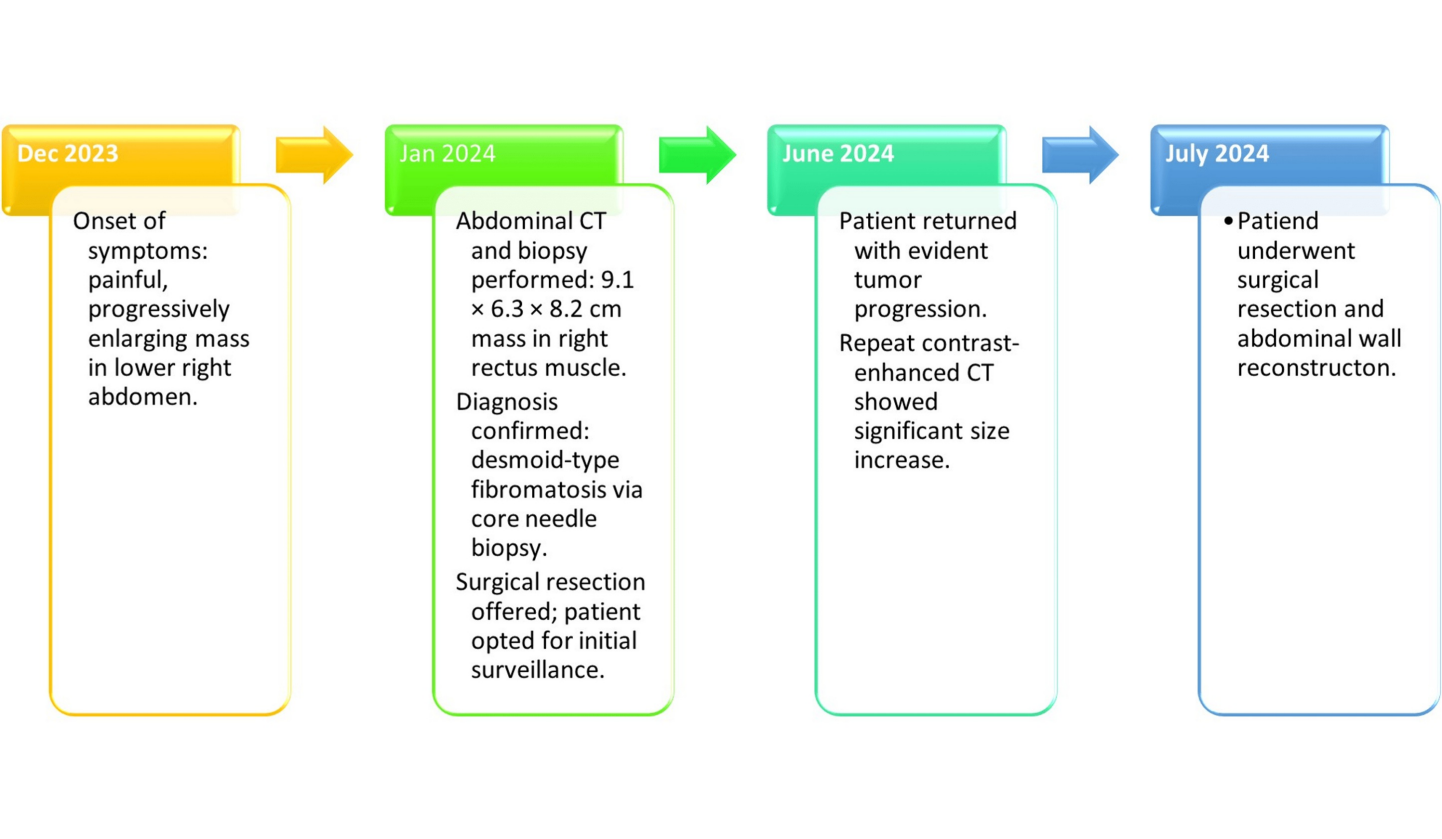
Clinical timeline of diagnosis and surgical management of a desmoid-type fibromatosis This timeline outlines the diagnostic and therapeutic course of a 37-year-old woman with a desmoid-type fibromatosis of the abdominal wall. The patient first presented in December 2023 with a painful, enlarging mass. Imaging and biopsy in January 2024 confirmed the diagnosis, but initial management was observational. By June 2024, tumor progression was evident on repeat imaging, prompting definitive surgical resection with abdominal wall reconstruction in July 2024.

We present the case of a 37-year-old woman with no significant past medical history. Her symptoms began in December 2023 with a painful, progressively enlarging mass in the lower right flank. She underwent clinical evaluation and abdominal CT with and without contrast, which revealed a mass arising from the right rectus abdominis muscle, measuring 9.1×6.3×8.2 cm. A trucut needle biopsy confirmed the diagnosis of desmoid-type fibromatosis. Surgical management was initially recommended; however, the patient declined intervention at that time. Six months later, she returned due to noticeable tumor growth (Figure 1). A repeat contrast-enhanced abdominal CT demonstrated a substantial increase in size (approximately double) of the desmoid tumor, still originating from the anterior rectus muscle (Figure 2). Given the rapid progression, the patient was scheduled for surgical resection with abdominal wall reconstruction (Figure 3).

**Figure 4:**
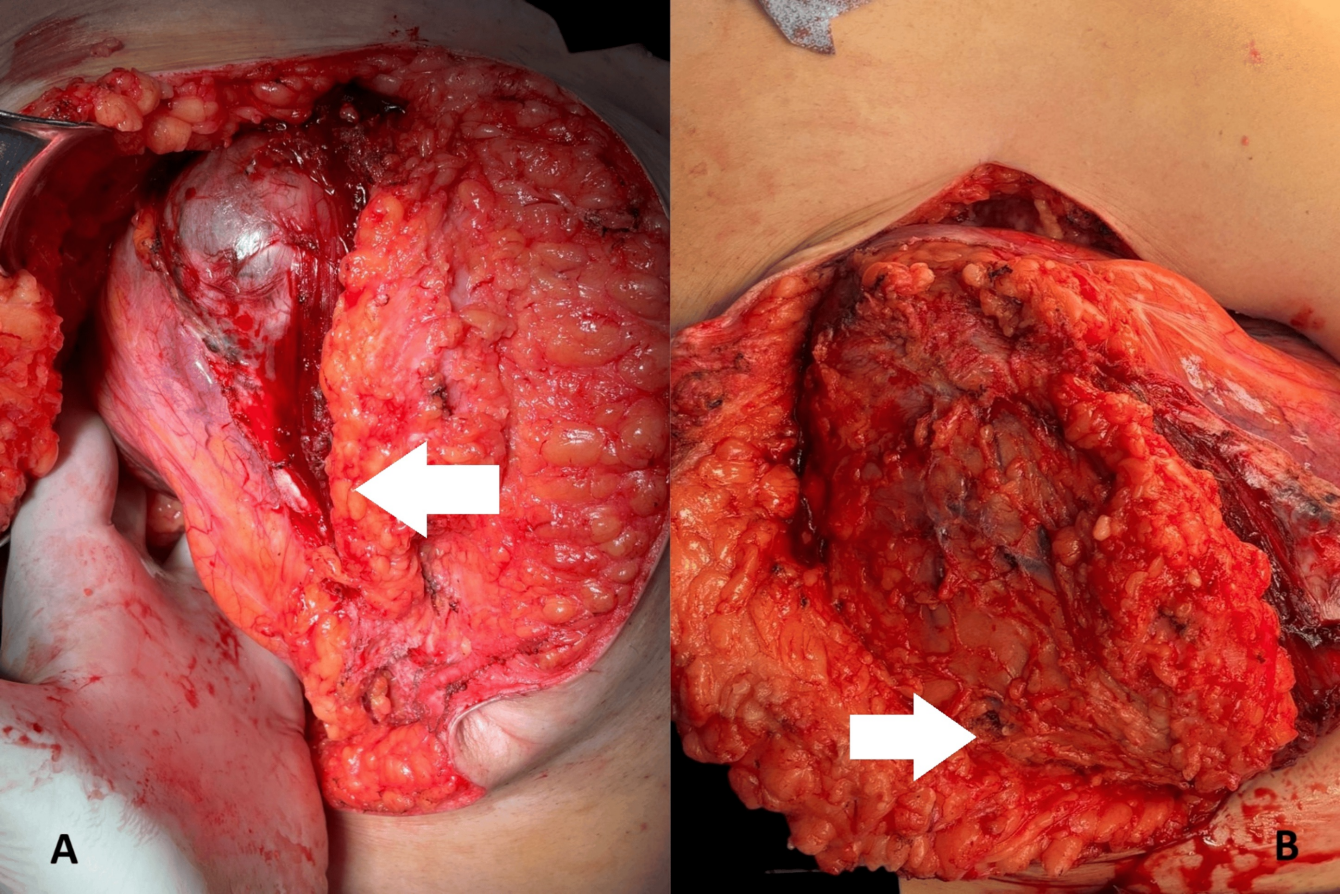
Intraoperative views during tumor resection (A) The mass is observed invading the right rectus abdominis muscle (white arrow), with dissection exposing the clear plane between the tumor and surrounding tissues; (B) The posterior surface of the resected mass (white arrow) shows infiltration into adjacent fascial layers and discoloration of underlying musculature.

**Figure 5:**
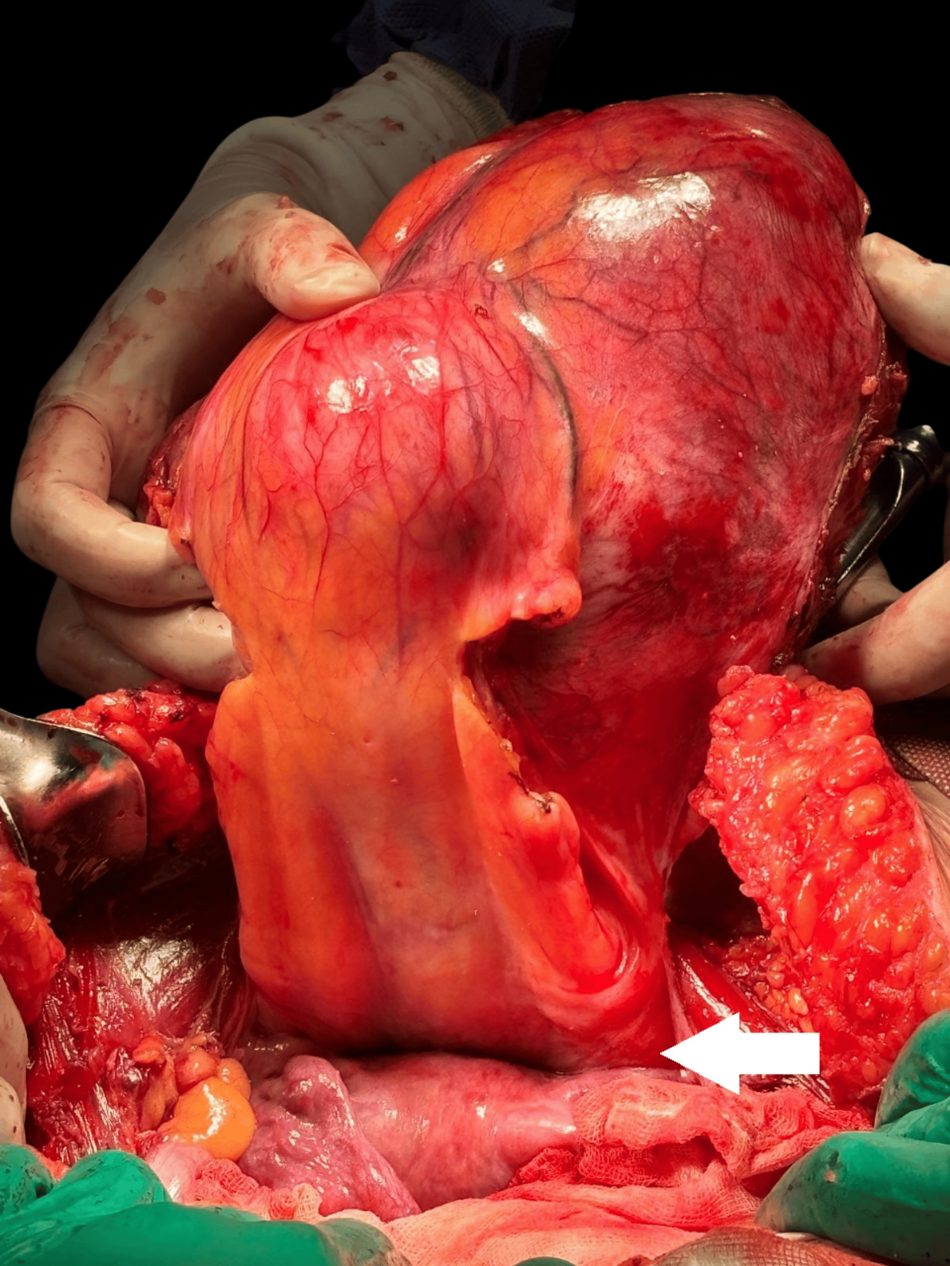
Gross intraoperative view showing the resected mass in relation to pelvic structures The uterus is identified and preserved (white arrow), located inferior to the tumor mass. Its orientation confirms the close anatomical relationship between the desmoid tumor and pelvic organs.

**Figure 6:**
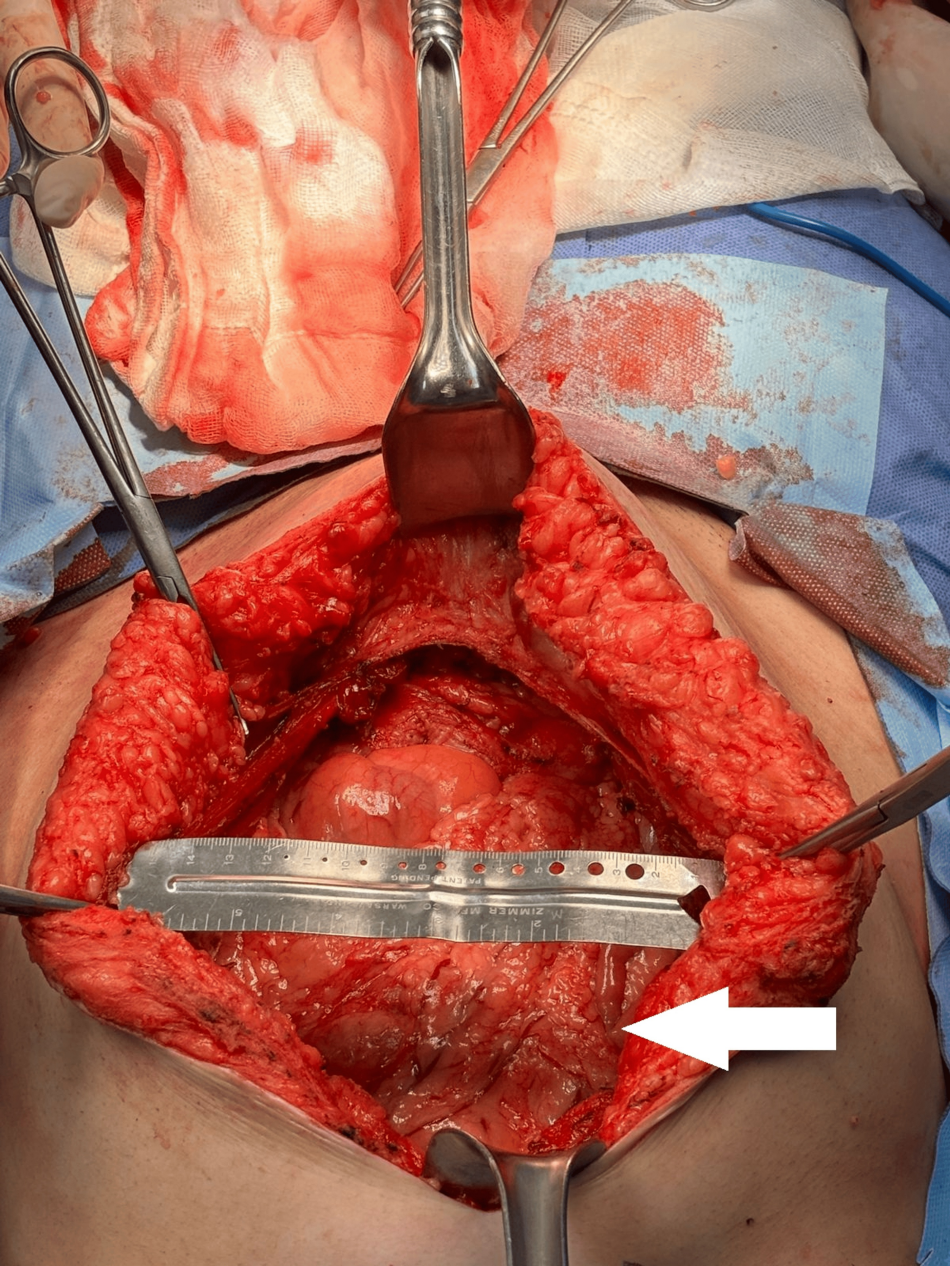
Intraoperative view of the abdominal wall defect following tumor resection The white arrow indicates the posterior fascia and peritoneal layer exposed after full-thickness excision of the tumor. The defect measures approximately 15x21 cm in width, with surrounding musculature and subcutaneous tissue retracted to demonstrate the full extent of resection.

**Figure 7:**
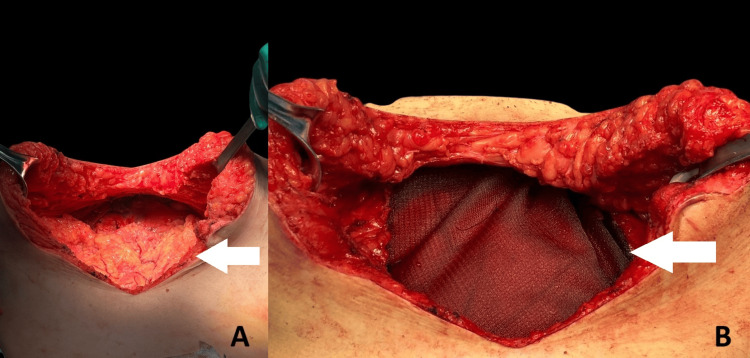
Reconstruction of the abdominal wall following tumor resection (A) Intraoperative view of the abdominal wall defect after tumor removal. The white arrow points to the posterior fascia, prepared for mesh placement; (B) Placement of a synthetic mesh over the defect (white arrow), secured to the fascial edges to ensure reinforcement of the abdominal wall.

A midline incision was made, proceeding with dissection through the layers of the abdominal wall until reaching the supra-aponeurotic plane. Dissection began at the left lateral margin of the mass, which was mobilized, revealing partial contact with the abdominal cavity. The tumor was further released from its lower pole, followed by careful dissection over the mass and beneath the right lateral subcutaneous tissue (Figure 4). During mobilization of the inferior region, the tumor was found to be in contact with the uterus (Figure 5). Complete excision of the mass was achieved, and the specimen was sent to the Pathology Department. Following resection, an abdominal wall defect measuring 15×21 cm was observed (Figure 6). The peritoneum and fascia were circumferentially released to facilitate reconstruction. The Sepramesh prosthesis was placed in a sublay position, anchored sequentially to the peritoneum and fascia, providing a tension-free, layered repair (Figure 7). A Blake drain was placed over the mesh and anchored using 2-0 nylon sutures. The subcutaneous tissue was closed in two layers with 1-0 and 2-0 vicryl sutures, and the skin was closed with skin staples. Estimated blood loss was approximately 50 cc.

**Figure 8:**
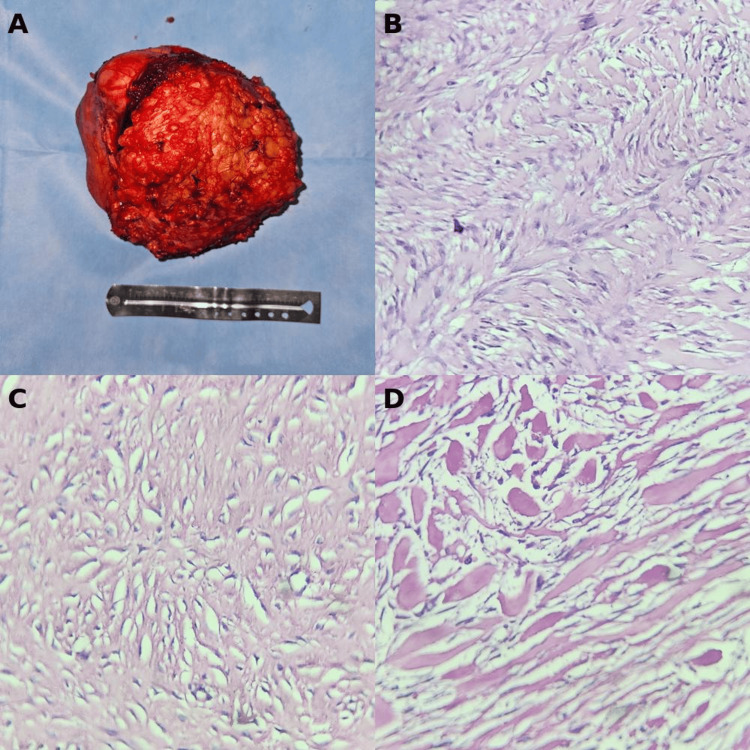
Surgical specimen and histopathological findings (A) Gross appearance of the resected tumor, displaying a well-circumscribed mass with adipose coverage, placed adjacent to a surgical ruler for scale; (B) Microscopic view showing a fascicular arrangement of spindle cells characteristic of fibromatosis; (C) Hypocellular area within the tumor, with sparse nuclei and abundant collagenous stroma; (D) Histologic section exhibiting features consistent with keloid-like change, including thick, hyalinized collagen bundles and low cellularity.

The final pathology report described the excised specimen as measuring 17×14×13.5 cm, with irregular shape and contours. The external surface was light brown and semi-firm to the touch. On sectioning, the tumor displayed a homogeneous, fibrous consistency, light brown coloration, and a characteristic swirling (storiform) pattern. Histological evaluation revealed a neoplasm composed of intersecting fascicles of spindle-shaped cells with elongated, bland-appearing nuclei and no cytologic atypia (Figure 8). Although the diagnosis was based on histomorphology, β-catenin IHC could provide confirmatory value when available. The tumor infiltrated the surrounding skeletal muscle fibers. No mitotic activity was observed. The report confirmed complete (R0) resection, with all margins free of residual tumor, a key prognostic factor in desmoid tumor surgery.

**Figure 9:**
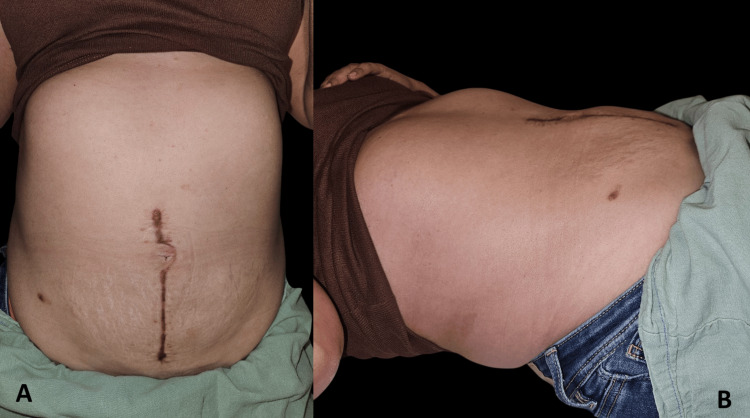
Postoperative follow-up showing aesthetic and structural outcomes (A) Frontal view of the abdomen at follow-up reveals a well-healed vertical midline scar, with no signs of dehiscence or hypertrophy; (B) Lateral view highlights preserved abdominal wall contour and volume symmetry, without evidence of herniation or bulging.

The patient was referred to physical rehabilitation, which she adhered to for a period of six months. At the eight-month postoperative follow-up, she reported being asymptomatic, with no limitations in mobility; the patient expressed high satisfaction with the aesthetic and functional outcomes. Clinical evaluation revealed appropriate wound healing, and both physical examination and follow-up ultrasound showed no evidence of tumor recurrence (Figure 9). The patient is undergoing clinical and ultrasound follow-up every 6 months. Annual MRI is planned given the risk of late recurrence, as supported by consensus recommendations.

## Discussion

Desmoid tumors (DTs), although histologically benign, present a distinct clinical challenge due to their locally aggressive behavior, potential for infiltration, and unpredictable natural history. Grossly, they appear as firm, irregular, gray-white fibrous masses aligned with muscle fibers, often mimicking scar tissue in both consistency and histological architecture [1]. Typically measuring between 5 and 20 cm, their size is a relevant prognostic factor and influences both treatment decisions and recurrence risk [7].

Histologically, DTs consist predominantly of collagen encasing uniform spindle-shaped cells with minimal mitotic activity. These tumors are poorly circumscribed and lack a true capsule, with frequent infiltration into adjacent soft tissue, muscle, and sometimes visceral organs. Peripheral inflammatory infiltrates, comprising macrophages, lymphocytes, and multinucleated giant cells, are frequently present [6,9].

Abdominal DTs may present as discrete palpable masses or as mesenteric plaques with diffuse infiltration, which can cause complications such as small bowel obstruction or vascular compromise due to progressive encasement and infiltration [1,7,10] (Table 1). Based on the Church classification, this case corresponds to stage IV due to the tumor's rapid growth and final size exceeding 20 cm, warranting urgent surgical resection. The histological and radiological presentation often contrasts, as these tumors may appear deceptively well-defined on imaging, despite ill-defined margins at the microscopic level [11].

**Table 1:** Church's desmoid tumor classification Source: Church et. al [10]

Stage	Clinical description	Recommended treatment
I	Asymptomatic, non-growing lesion	Observation or prophylactic therapy with NSAIDs. Surgical resection only if incidentally discovered during another procedure.
II	Symptomatic and growing, <10 cm in diameter	Surgical resection if feasible. If unresectable, initiate combination medical therapy (e.g., tamoxifen or raloxifene plus NSAID).
III	Symptomatic (10–20 cm) or asymptomatic but slowly growing lesion	Active treatment indicated. Options include NSAIDs, tamoxifen, raloxifene, vinblastine, methotrexate, and anthracycline-based chemotherapy (e.g., doxorubicin).
IV	Symptomatic, >20 cm or rapid growth, or presenting complications	Urgent, often aggressive therapy, including extensive surgical resection, chemotherapy (e.g., anthracycline-based), and radiotherapy as needed.

Management strategies for DTs have evolved significantly in recent years. Historically, radical surgical resection was the mainstay of treatment; however, recognition of the high recurrence rates even with clear margins and the potential morbidity associated with surgery has led to a paradigm shift. Current consensus favors a risk-adapted, conservative approach, particularly in asymptomatic or stable patients, with active surveillance being a valid option in many cases [8].

When intervention becomes necessary-due to progressive symptoms, anatomical risk, or tumor growth-treatment modalities must be tailored to tumor location, resectability, and the patient's overall condition. Surgery is reserved for symptomatic lesions causing functional impairment or those threatening vital structures. In such cases, the objective is complete (R0) resection with negative margins, although R1 margins may be accepted if function or cosmesis is at risk [8]. Nevertheless, incomplete resections increase recurrence risk, particularly in tumors exceeding 5 cm [11].

Desmoid tumors of the abdominal wall pose unique reconstructive challenges. Full-thickness resections often necessitate extensive parietal reconstruction, and when the peritoneum or adjacent organs are involved, en bloc resection is recommended [5,11]. Several reconstructive techniques have been described, including fascial grafts, myocutaneous flaps (e.g., rectus abdominis, tensor fascia lata), and synthetic mesh placement. Ensuring a vascularized and aseptic reconstruction is paramount, particularly when meshes are used in contact with intra-abdominal contents [5,12]. In this context, mesh-reinforced reconstruction has demonstrated favorable outcomes. Zhao et al. recently reported a retrospective analysis of 16 patients undergoing abdominal wall desmoid tumor resection with mesh repair, showing low complication rates, no incidences of hernia or bulging, and durable outcomes over long-term follow-up. Their findings support the feasibility and safety of mesh-based approaches when performed with careful surgical planning and technique [13].

Radiation therapy is a valid adjunct or alternative for patients unfit for surgery or when surgical morbidity is high. Although its role in intra-abdominal DTs remains limited due to proximity to radiosensitive structures, moderate-dose radiotherapy has shown efficacy in selected cases with progression or persistent symptomatic disease [8]. Likewise, hormonal influences, such as pregnancy and estrogen exposure, suggest a potential role for medical management with anti-estrogen therapies in specific scenarios [6].

Despite their aggressive local behavior, DTs do not metastasize. Nonetheless, their clinical course is variable-ranging from spontaneous regression to relentless growth. This heterogeneity necessitates individualized therapeutic planning, guided by a multidisciplinary team and informed by tumor behavior, anatomical constraints, and patient quality of life [7,14]. Our management approach aligns with current recommendations by the Desmoid Tumor Working Group (2020) [15], which endorse active surveillance as first-line therapy for stable or asymptomatic DTs, reserving surgical intervention for cases demonstrating significant progression, symptomatic burden, or anatomical compromise. In this case, rapid tumor enlargement and associated symptoms justified the decision to proceed with definitive surgical resection.

## Conclusions

Surgical management remains a cornerstone in the treatment of desmoid tumors when rapid progression, mass effect, or anatomical compromise necessitate intervention. This case underscores the critical role of timely resection and meticulous abdominal wall reconstruction in achieving oncologic control while preserving structural integrity and function. Complete (R0) excision was achieved, which is a key prognostic factor in minimizing local recurrence. While the initial approach was conservative, consistent with current guidelines, the transition to surgery was warranted due to the tumor's rapid progression and increasing symptom burden, highlighting how clinical behavior may necessitate deviation from prolonged observation or systemic therapy.

The use of mesh-reinforced reconstruction in this case not only provided a structurally sound and aesthetically favorable outcome but also aligns with recent literature demonstrating its safety and long-term durability in similar scenarios. Given the unpredictable nature of desmoid tumors, ongoing surveillance with clinical and imaging follow-up remains essential to ensure early detection of recurrence. Ultimately, this case reinforces the importance of individualized, guideline-informed decision-making and multidisciplinary collaboration in the management of desmoid-type fibromatosis, especially when involving critical anatomical regions like the abdominal wall.
